# Likelihood of childbirth in women with one versus two ovaries: a Swedish population-based study of women treated with unilateral oophorectomy for benign indications

**DOI:** 10.1093/hropen/hoag041

**Published:** 2026-05-25

**Authors:** Tekla Lind, Hanna P Nilsson, Mikael Andersson Franko, Kenny A Rodriguez-Wallberg

**Affiliations:** Department of Reproductive Medicine, Division of Gynaecology and Reproduction, Karolinska University Hospital, Stockholm, Sweden; Department of Clinical Science and Education, Södersjukhuset, Karolinska Institutet, Sweden, Stockholm; Laboratory of Translational Fertility Preservation, Department of Oncology-Pathology, Karolinska Institutet, Stockholm, Sweden; Department of Clinical Science and Education, Södersjukhuset, Karolinska Institutet, Sweden, Stockholm; Department of Reproductive Medicine, Division of Gynaecology and Reproduction, Karolinska University Hospital, Stockholm, Sweden; Laboratory of Translational Fertility Preservation, Department of Oncology-Pathology, Karolinska Institutet, Stockholm, Sweden

**Keywords:** childbirth, infertility, ovarian surgery, parity, population-based register study, pregnancy, unilateral oophorectomy

## Abstract

**STUDY QUESTION:**

What is the likelihood of future childbirth in women undergoing unilateral oophorectomy (UO) for benign indications, when compared to age-matched women with intact ovaries?

**SUMMARY ANSWER:**

A significantly reduced likelihood of future childbirth was observed in women with a history of UO, when compared to women with intact ovaries.

**WHAT IS KNOWN ALREADY:**

At the time of this study, the prevailing view is that a single ovary is sufficient to maintain fertility. This is the first large-scale observational study reporting an association between UO for benign indications and a decreased chance of future childbirth in a population-based cohort that was followed until the end of reproductive age.

**STUDY DESIGN, SIZE, DURATION:**

This population-based register study included all Swedish women born 1955–1966, followed until the end of female reproductive age. We identified 17 856 women who underwent UO (exposed) and 171 731 age-matched controls. After exclusions, the exposed cohort consisted of 10 469 women of fertile age with a benign indication for UO and 101 753 age-matched controls.

**PARTICIPANTS/MATERIALS, SETTING, METHODS:**

All cases of UO were identified by surgery codes and diagnosis of benign diseases, and up to 10 controls per case, matched by age and county, were used in the analysis. Childbirth data were collected from the Swedish Medical Birth Register, and childbirth rates were compared between exposed cases and age-matched controls.

**MAIN RESULTS AND THE ROLE OF CHANCE:**

The childbirth rate post-surgery was lower in women with UO compared to age-matched controls having intact ovaries: 25.5% versus 28.7%, respectively (risk ratio (RR) 0.89; confidence interval (95% CI) 0.86–0.91). Subgroup analysis of women who were nulliparous at the time of UO (N = 4083) versus their age-matched controls (N = 18 770) also indicated a lower childbirth rate: 41.3% vs 66%, respectively (RR 0.63; 95% CI 0.61–0.65). Additionally, an interaction analysis among the nulliparous women indicated that the reduced likelihood of having children was associated with older age at the time of UO.

**LIMITATIONS, REASONS FOR CAUTION:**

In this register-based study, the desire for pregnancy could not be captured, and it was unknown if it was similar among cases and controls. Although malignant indications of UO could be excluded in the study, the histopathology of the removed ovaries was not available; thus, it was not possible to adjust for specific diseases potentially affecting fertility, and therefore, residual confounding is a limitation. Additional limitations included the lack of adjustment for lifestyle factors, as such data was only available from the medical birth register, and thus only accessible for the women who had given birth.

**WIDER IMPLICATIONS OF THE FINDINGS:**

This large population-based study demonstrates a significant association between unilateral oophorectomy for benign indications and a reduced likelihood of subsequent childbirth; however, causality cannot be inferred from these observational data.

**STUDY FUNDING/COMPETING INTEREST(S):**

This work was funded by the Swedish Cancer Society (20 0170 F), the Swedish Research Council (Dnr 2021-06116 and Dnr 2023-01872), the Radiumhemmets Research Funds Grant for clinical researchers 2020–2026, the Childhood Cancer Fund (Dnr PR2022-0081), the Stockholm County Council (FoUI-953912) and the Karolinska Institutet Research grants Dnr 2020-01963 to K.A.R.W.

The funders of the study had no role in study design, data collection, data analysis, data interpretation, or writing of the report. The authors had full access to all study data and the final responsibility for the decision to submit for publication.

K.A.R.W. has received: research grants from NovoNordisk, Merck, Gideon Richter and Ferring Pharmaceuticals; royalty for Student Literature (medical text book), Sweden; consulting fees from the Swedish Ministry of Health and Welfare as expert in assisted reproduction and fertility preservation for transgender people; consulting fees from SpringWorks; honoraria from Roche and Pfizer for chairmanship and lectures for education of oncologists; honoraria from Organon for lectures for ob/gyn and reproductive medicine specialists; and honoraria from IBSA for Advisory Board for educational events. K.AR.W. has also received support from Organon for participation in Journées Fertilité 2023, Paris, France, and support from Region Stockholm for participation as chair of the Swedish Interest Group in Reproductive Medicine Fert-ARG, for the Swedish Society of Obstetrics and Gynecology SFOG; participates at advisory board for Merck, Nordic countries, and on an advisory board for Ferring, National coordinator of study; and has received Time-lapse equipment for pre-clinical research from Merck pharmaceuticals and a grant for experimental *in vitro* research from Ferring Pharmaceuticals. The remaining authors have no conflicts of interest to declare.

**TRIAL REGISTRATION NUMBER:**

N/A

WHAT DOES THIS MEAN FOR PATIENTS? Some women need to have one ovary removed because of non-cancerous conditions, such as cysts or pain. For a long time, doctors have believed that having one remaining ovary is enough to maintain the ability to have children. However, there has been limited research to confirm this.

In this study, we examined whether women who had one ovary removed were as likely to have children later in life as women of the same age who did not have surgery on their ovaries. Using population-based data and long-term follow-up until the end of female reproductive age, we compared childbirth outcomes between these two groups.

We found that women who had one ovary removed were significantly less likely to give birth in the future compared with women who had both ovaries intact. This study is the first large population-based analysis to show a link between the removal of one ovary for benign reasons and a reduced chance of having children later in life.

These findings suggest that removing one ovary may have a greater impact on future fertility than previously thought, and highlight the importance of considering long-term reproductive outcomes when planning ovarian surgery.

## Introduction

Unilateral oophorectomy (UO) is the surgical removal of one ovary. The most common underlying diagnosis leading to the intervention is a benign ovarian cyst, but UO is also indicated for treatment of malignant ovarian tumors, endometriosis, tubo-ovarian abscesses, and ovarian torsion (Lawson and Rentea, 2022).

The effects of a unilateral oophorectomy (UO) on fertility and menopause are not well studied, and the prevailing view, that a single ovary can be sufficient to maintain fertility and hormone homeostasis, is only an assumption (Gasparri *et al.*, 2021). In previous studies, UO, with loss of half the anatomical ovarian reserve, has been associated with a small shortening of women’s reproductive life span, advancing menopausal age only by 1–2 years (Hardy and Kuh, 1999; Yasui *et al.*, 2012; Bjelland *et al.*, 2014; Rosendahl *et al.*, 2017), with an earlier onset of perimenopausal symptoms (Hardy and Kuh, 1999). A previous report of earlier age at menopause in association with younger age at UO suggested that an early UO could have a higher impact on future reproductive lifespan (Rosendahl *et al.*, 2017). With regards to natural pregnancy rate after UO, it has been suggested that UO might have a greater impact if the woman is older, or if their ovarian reserve is reduced, but generally the procedure to remove one ovary surgically has not been considered as an important factor affecting fertility potential (Lass, 1999; Gnoth *et al.*, 2003; Bellati *et al.*, 2014; Vasconcelos and de Sousa Mendes, 2015; Lawson and Rentea, 2022).

In ART treatments, exogenous gonadotropins are used to stimulate multiple follicle recruitment and maturation of oocytes. Since an increased dose of gonadotropins will improve oocyte yields even after UO, it has been proposed that the remaining ovary might work to compensate for the removal of the contralateral one (Lass *et al.*, 1997; Khan *et al.*, 2014). However, a large multicentric case-controlled study of women with UO undergoing ART versus controls has shown a significantly reduced live birth rate in women with previous UO compared with those with intact ovaries (Lind *et al.*, 2018). These results are supported by a recent meta-analysis of all studies published until 2021 (Rodriguez-Wallberg *et al.*, 2022).

To investigate the fertility outcomes following the removal of one ovary during reproductive age, we designed a study using the Swedish population-based registers, with the objective of determining the likelihood of future childbirth in women who underwent UO, compared to age-matched women with intact ovaries.

## Materials and methods

### Ethical approval

Approval from the Regional Ethics Committee in Stockholm, Sweden (Dnr-2013/2287-31/4) was obtained prior to study initiation.

### Study population and data sources

This population-based register study was conducted using nationwide Swedish health registers with prospectively collected data. All residents of Sweden are assigned a unique personal identification number at birth or immigration, which enables accurate linkage of individual-level data across national registers.

The registers used in this study have mandatory reporting, high validity, and population coverage exceeding 98%. Register quality is continuously monitored by the Swedish authorities, and linkage between registers has been shown to be highly reliable, minimizing the risk of misclassification or incomplete outcome ascertainment.

In this population-based register cohort study, all women born in Sweden between 1955 and 1966 who underwent a UO for benign conditions before the age of 46 were identified as exposed (cases) through the records from the National Patient Register (NPR) (Ludvigsson *et al.*, 2011). Cases were identified, including diagnosis codes for unilateral oophorectomy, laparoscopic unilateral oophorectomy, unilateral salpingo-oophorectomy, and laparoscopic unilateral salpingo-oophorectomy (7020, 7023, 7030, 7033, LAE10, LAE11, LAF00, and LAF01).

An unexposed control cohort of age-matched women without such a surgical diagnosis was obtained from the Total Patient Register (TPR). In addition to age matching, the controls were also matched by county of residence at the time of the index date, if possible, at a case-to-control ratio of 1:10. The controls were followed from the index date of their matched case. Both cases and controls were followed until the end of the natural reproductive lifespan, determined as 46 years of age or death.

The index date was defined as the date of unilateral oophorectomy for each exposed woman. For unexposed controls, follow‑up commenced on the same index date as their matched case, corresponding to the date of oophorectomy. Secondary outcomes were identified through their corresponding ICD codes (Supplementary Table S1).

Hospitalizations, surgical information, and data from non-primary care outpatient visits were all coded according to the Swedish version of the International Classification of Diseases (ICD–SE) and the Classification of Surgical Procedures (Nordic Medico-Statistical Committee). Three different editions of ICD codes were used during the study period: the 8th, 9th, and 10th editions. Information on cases and controls was assembled, using cross-linked data from the Swedish healthcare quality registers. To exclude malign indications, information on all gynaecological cancers was retrieved from the NPR and the Swedish Cancer Register (SCR), where health care providers in Sweden report new cancer cases. Reporting to the SCR has been mandatory since 1958, and the register has nearly 98% morphologically verified cases (National Board of Health and Welfare, 2011). The Cause of Death Register (CDR), which has logged all deaths and their causes since 1961, was used to collect information on the date of death.

The primary outcome was childbirth, defined as at least one live birth after the index date, identified through the Medical Birth Register (MBR). This register captures all live births in Sweden regardless of mode of conception. The MBR includes data from early pregnancy to postpartum care on more than 98% of all births since 1973 (National Board of Health and Welfare, 2003). The main outcome measure was childbirth, which was dichotomized into ‘yes’ or ‘no’ for at least one child in the data obtained from the MBR. Data on deliveries occurring before the index were used to identify the women who were parous at the index date. Age at time of index was grouped into ≤ 25, 26–35, and 36–45.

The Swedish register of Education (SRE) was used to retrieve information on the highest achieved educational level during the study period; this data was used as a proxy for socioeconomic status. Data on female infertility, surgery for female infertility, and surgical or medical treatment for extrauterine pregnancy and pregnancy through IVF were obtained from the NPR, which has registered data about IVF treatment since 1981, when the first IVF was performed in Sweden.

The secondary outcome of infertility was defined as a clinically registered infertility diagnosis in the National Patient Register, which in Swedish healthcare practice is assigned after ≥12 months of unsuccessful attempts at conception followed by a fertility work-up indicating the need for investigation and/or treatment.

Endometriosis was identified using registered ICD-8, ICD-9, and ICD-10 diagnostic codes in the National Patient Register. Diagnoses recorded prior to the index date or up to eight weeks after the index date were included to capture clinically recognized disease potentially related to the indication for unilateral oophorectomy. Histopathological confirmation of endometriosis was not available in the registers.

**Figure 1.**
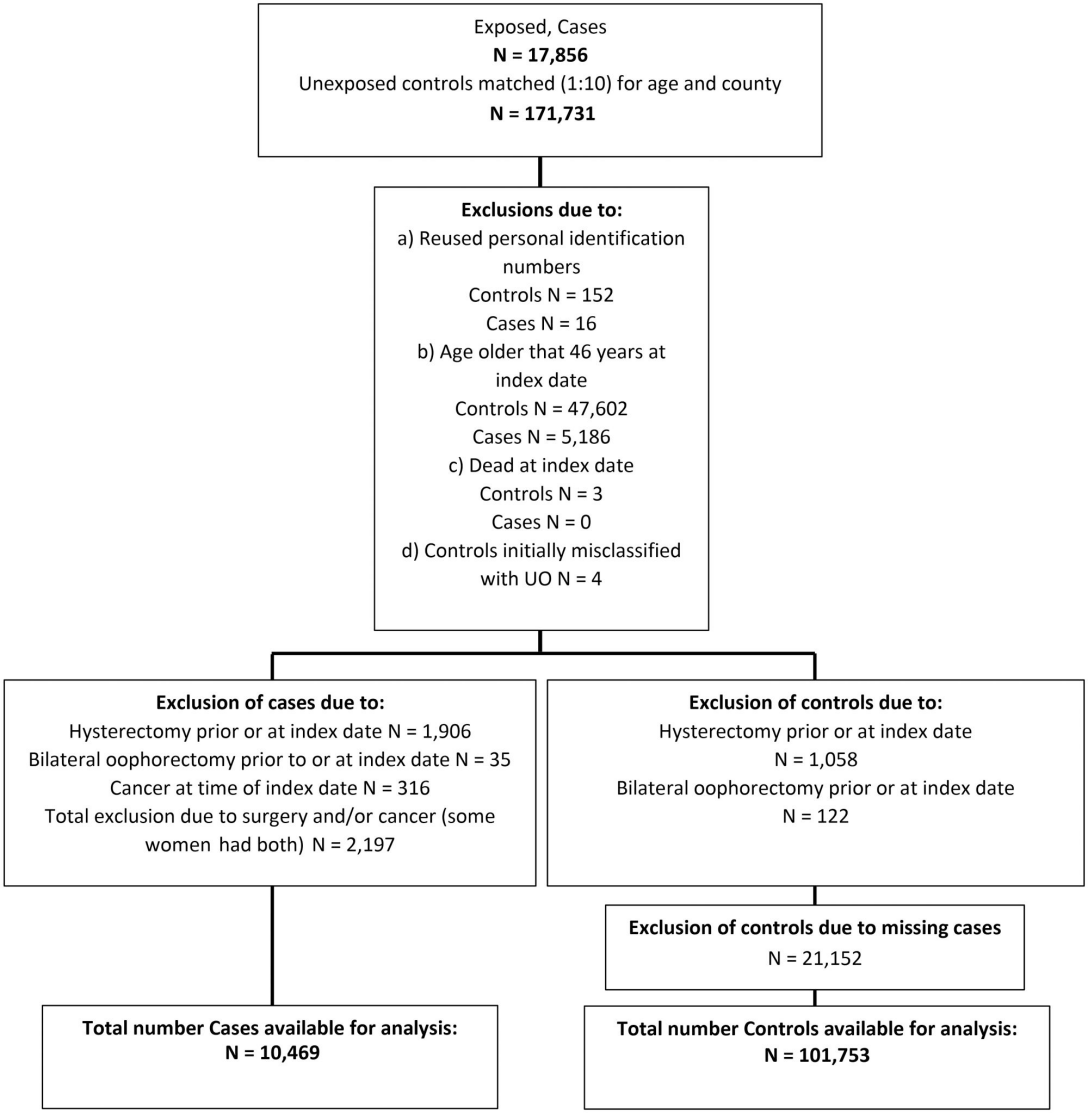
**Study flowchart.** Flow diagram showing the derivation of the final analytic cohort after sequential exclusions, with exposed cases and age- and county-matched unexposed controls (1:10). *Note*: some cases or controls were excluded for more than one reason.

All women undergoing a hysterectomy or bilateral oophorectomy prior to, or at, the index date were excluded. Cases and controls were also excluded based on death or age above 46 prior to the index. Exposed cases were excluded if they were diagnosed with cancer within two months of their UO. Finally, all controls without matched cases were excluded (Fig. 1).

### Statistical analysis

Differences in the study population characteristics were tested with a chi-square test. Parity in women with a history of UO was compared to that of matched controls using a Generalized Estimating Equation (GEE) model with binomial distribution and a log link to present Risk Ratios (RR) and a sandwich estimator to account for possible clustering within matched pairs. The remaining dichotomous outcomes were analysed in a similar manner.

A subgroup analysis was done for women who were nulliparous before surgery and their nulliparous age-matched controls. Sensitivity analyses were performed: (i) with exclusion of cases and controls with registered infertility treatments prior to index; (ii) with exclusion of all women with a diagnosis of endometriosis prior to or up until eight weeks post index; (iii) with exclusion of women where any of the following events occurred post index but before the age of 46 years: hysterectomy, bilateral oophorectomy, a second unilateral oophorectomy, or death; and (iv) with exclusion of all women above the age of 35 at index.

To investigate whether the association between UO and parity after surgery differed depending on age, the interaction effect between age group and UO was investigated; this provided the additional advantage of being able to test for an age-modifying effect.

All analyses were performed in R version 4.2.3 (R Foundation for Statistical Computing, Vienna, Austria), with a significance set at 5%. All *P*-values and confidence intervals were two-sided.

## Results

### Sample characteristics

We identified 17 856 exposed women matched with 171 731 controls. After exclusions, the exposed cohort consisted of 10 469 women of fertile age with a benign indication for unilateral oophorectomy and their 101 753 age-matched controls (Fig. 1).

Table 1 shows the age and demographics of the cohort at the index date, as well as the time period for the UO. Most women undergoing UO were older than 35 at index. Women with a history of UO had a lower highest lifetime achieved educational level. The exposed cases also had a lower parity before index (61.0% vs 66.1%); for the subgroup analysis of the nulliparous women, among the older cases, their age-matched controls were more often parous prior to the index date, and thus the ratio of controls to cases was lower in this subgroup analysis (Table 1). Women were excluded from the sensitivity analyses due to: post-index hysterectomy in 11% of cases compared to 4.1% of controls (*P* ≤ 0.001), post-index oophorectomy in 9.5% of cases vs 1.4% of controls (*P* ≤ 0.001), death before age 46 in 4.1% of cases vs 1.8% of controls (*P* ≤ 0.001), endometriosis in 18.4% of cases vs 0.3% of controls (*P* ≤ 0.001) and previous infertility in 6.0% of cases vs 3.5% of controls (*P* ≤ 0.001) (Supplementary Table S2).

**Table 1.** Sample characteristics at index date.

	All women			Nulliparous women prior to index date		
	N = 112 222			N = 22 853		
Variable	Cases with UO	Controls	*P*-value^2^	Cases with UO	Controls	*P*-value^2^
	(N = 10 469) (%)	(N = 101 753) (%)		(N = 4083) (%)	(N = 18 770) (%)	
**Age at index date**			0.53 ^ns^			<0.0001***
≤25	1710 (16.3%)	17 046 (16.8%)		1374 (33.7%)	11 432 (60.9%)	
26–35	3470 (33.1%)	33 695 (33.1%)		1360 (33.3 %)	4910 (26.2%)	
≥36	5289 (50.5%)	51 012 (50.1%)		1349 (33.0%)	2428 (12.9%)	
**Highest educational level** *			<0.0001***			<0.0001***
Below high school	1560 (15.1%)	12 473 (12.4%)		551 (13.9%)	1882 (10.1%)	
High school	5449 (52.8%)	52 420 (51.9%)		1943 (49.0%)	9235 (49.7%)	
Beyond high school	3320 (32.1%)	36 031 (35.7%)		1475 (37.2%)	7461 (40.2%)	
**Index date by time period**			0.65 ^ns^			<0.0001***
1972–1981	892 (8.5%)	8873 (8.7%)		736 (18.0%)	6402 (34.1%)	
1982–1991	2800 (26.7%)	27 500 (27.0%)		1475 (36.1%)	8306 (44.3%)	
1992–2001	4255 (40.6%)	40 742 (40.0%)		1272 (31.2%)	3005 (16.0%)	
2002–2012	2522 (24.1%)	24 638 (24.2%)		600 (14.7%)	1057 (5.6%)	
**Children before index date**			<0.0001***			
Yes	6386 (61.0%)	67 240 (66.1%)				
No	4083 (39.0%)	34 513 (33.9%)				

* Highest educational level achieved up to 45 years of age.

2 Significance as indicated by a chi-square test ns: *P* > 0.05 (not significant); *: *P* ≤ 0.05 (significant); **: *P* ≤ 0.01 (highly significant); ***: *P* ≤ 0.001 (highly significant).

UO, unilateral oophorectomy.

### Primary and secondary outcomes

Among the women with a history of UO, 25.5% had one or more children after surgery, compared to 28.7% among the matched controls (RR 0.89; 95% CI 0.86–0.91). The subgroup analysis of nulliparous women undergoing UO indicated a 37% lower chance of having a child post index (RR 0.63; 95% CI 0.61–0.65), when compared to the nulliparous controls (41.3% vs 66%, respectively; Table 2). The number of children post index was also decreased among the exposed nulliparous cases, where 32.4% of the exposed cohort had one or two children and 8.9% had three children or more, compared to 50.1% and 15.9% respectively among matched nulliparous controls (*P* ≤ 0.001) (Supplementary Table S2). Women with a history of UO also had a higher risk of infertility, surgery for infertility, and IVF treatment. This difference was significant among the nulliparous women, but the results were more pronounced among the total cohort (Table 2).

**Table 2.** Association between unilateral oophorectomy and parity (live birth).

	All women N = 112 222				Nulliparous women prior to index N = 22 853			
Variable	Cases with UO	Controls	RR (95% CI)	*P*-value^2^	Cases with UO	Controls	RR (95% CI)	*P*-value^2^
	N = 10 469 (%)	N = 101 753 (%)			N = 4083 (%)	N = 18 770 (%)		
One or more children born	2665 (25.5)	29 168 (28.7)	0.89 (0.86 to 0.91)	<0.0001***	1687 (41.3)	12 389 (66.0)	0.63 (0.61 to 0.65)	<0.0001***
Age at index <25 years	1246/1710 (72.9)	13 857/17 046 (81.3)	0.90 (0.87 to 0.92)	<0.0001***	1012/1374 (73.6)	9467/11 432 (82.8)	0.89 (0.86 to 0.92)	<0.0001***
Age at index 26–35 years	1255/3470 (36.2)	13 449/33 695 (39.9)	0.91 (0.87 to 0.95)	<0.0001***	617/1360 (45.4)	2749/4910 (56.0)	0.81 (0.76 to 0.86)	<0.0001***
Age at index >36 years	164/5289 (3.1)	1862/51 012 (3.6)	0.85 (0.73 to 0.99)	0.039*	58/1349 (4.3)	173/2428 (7.1)	0.60 (0.45 to 0.81)	0.0007***
Female infertility^b^	598 (5.7)	3367 (3.3)	1.73 (1.59 to 1.87)	<0.0001***	446 (10.9)	1584 (8.4)	1.29 (1.17 to 1.43)	<0.0001***
Surgery for infertility	134 (1.3)	507 (0.5)	2.57 (2.13 to 3.10)	<0.0001***	106 (2.6)	279 (1.5)	1.75 (1.40 to 2.17)	<0.0001***
IVF treatment^a^	44 (0.4)	165 (0.2)	2.59 (1.86 to 3.61)	<0.0001***	40 (1.0)	81 (0.4)	2.27 (1.56 to 3.31)	<0.0001***

Association between unilateral oophorectomy (UO) and parity **(**live birth) in the whole cohort and in the subgroup of women who were nulliparous at the time of UO. Infertility diagnosis, surgery for infertility, and performance of infertility treatments with IVF post-index are also indicated in each group.

a IVF treatments only women with an index date from 1980 onwards.

b Clinically registered infertility.

2 Significance as determined by a Generalized Estimating Equation (GEE) model with binomial distribution indicated by ns: *P* > 0.05 (not significant); *: *P* ≤ 0.05 (significant); **: *P* ≤ 0.01 (highly significant); ***: *P* ≤ 0.001 (highly significant).

### Sensitivity analyses

The differences between cases and controls remained after the sensitivity analyses, both after excluding death, secondary hysterectomy, bi- or unilateral oophorectomy post-index, and after excluding all women above 35 years of age at index (Supplementary Table S3). Removal of the oldest age group did not have a marked effect on the full cohort, but among nulliparous women, it reduced the comparative risk of not having further children from RR 0.63 (95% CI 0.61–0.65) to RR 0.80 (95% CI 0.77–0.82; Supplementary Table S4). Similarly, the subgroup analysis of age at time of index indicated that the likelihood of childbirth was significantly reduced in all age categories, both in the full cohort and among the nulliparous women (Table 2). But among the nulliparous women, the observed effect of UO on childbirth was in part dependent on older age at the time of the surgery (Table 2). An interaction analysis showed a significant difference between the age categories (*P* = 0.002).

In the cohort undergoing UO, 1924 of 10 469 women (18%) had a diagnosis of endometriosis. Among the controls, we identified a total of 327 (0.003%) women diagnosed with any type of endometriosis up until 8 weeks post-index. After exclusion of these cases and their matched controls, the women who underwent UO still had a significantly reduced chance of childbirth (RR 0.89; 95% CI 0.87–0.92) (Table 3). Thus, while exclusion of women with endometriosis affected the absolute numbers undergoing treatment for infertility after UO, it did not decrease the risk among the cases (Table 3). Exclusion of women with previous treatment for infertility similarly did not affect the likelihood of childbirth post index (all women RR 0.86 (95% CI 0.83–0.88), nulliparous women RR 0.62 (95% CI 0.60–0.64)) and only increased the comparative risks for later infertility or infertility treatment among the remaining cases (Supplementary Table S5).

**Table 3.** The association between unilateral oophorectomy and parity (live birth) after exclusion for endometriosis.

	All women N = 91 361				Nulliparous women prior to index N = 18 819			
Variable	Cases with UO	Controls	RR (95% CI)	*P*-value^2^	Cases with UO	Controls	RR (95% CI)	*P*-value^2^
	N = 8545 (%)	N = 82 816 (%)			N = 3139 (%)	N = 15 680 (%)		
One or more children born	2306 (27.0)	25 079 (30.3)	0.89 (0.87–0.92)	<0.0001***	1423 (45.3)	10 808 (68.9)	0.66 (0.64–0.68)	<0.0001***
Female infertility	470 (5.5)	2916 (3.5)	1.56 (1.43–1.71)	<0.0001***	341 (10.8)	1366 (8.7)	1.25 (1.12–1.39)	0.0001***
Surgery for infertility	101 (1.2)	438 (0.5)	2.23 (1.81–2.77)	<0.0001***	80 (2.6)	236 (1.5)	1.69 (1.32–2.17)	<0.0001***
IVF treatment^a^	32 (0.4)	140 (0.2)	2.22 (1.51–3.24)	<0.0001***	28 (0.9)	65 (0.4)	2.15 (1.39–3.34)	0.0006***

The association between unilateral oophorectomy (UO) and parity (live birth) was investigated after exclusion of women with endometriosis, before 8 weeks after the index date. Infertility diagnosis, surgery for infertility, and performance of infertility treatments with IVF post-index are also indicated in each group.

a IVF treatments only women with an index date from 1980 onwards.

2 Significance as indicated by a Generalized Estimating Equation (GEE) model with binomial distribution, ns: *P* > 0.05 (not significant); *: *P* ≤ 0.05 (significant); **: *P* ≤ 0.01 (highly significant); ***: *P* ≤ 0.001 (Highly significant).

## Discussion

This large population-based study of all women born in Sweden 1955–1966 undergoing a unilateral oophorectomy for benign indications during the fertile age found a significantly lower likelihood of post-surgery childbirth (RR 0.89; 95% CI 0.86–0.91) in women with UO, when compared to age-matched controls with intact ovaries. The observed differences between cases and controls were increased among the subgroup of women who were nulliparous at the time of UO, and the risk of not achieving livebirth also increased with increasing age at index date. The women undergoing UO had a higher risk of post-index infertility diagnoses, surgery for infertility, and IVF treatments. Although assisted reproductive technologies are more widely used today than during the early study period, the present findings remain relevant for counselling women undergoing unilateral oophorectomy, as many women still rely on natural conception, and fertility preservation of ovarian tissue remains a key surgical consideration. Sensitivity analyses were performed for women with a diagnosis of endometriosis or with previous fertility treatments, and the exclusion of those cases did not significantly affect the results. While exclusion of women with endometriosis affected the absolute numbers undergoing treatment for infertility after UO, it did not decrease the risk among the cases. Age and underlying infertility might in part explain the observed effect, but the reductions in childbirth rate after UO were, in all cases, significant.

Limited information is available on natural pregnancy after UO in the literature (Lass, 1999; Gnoth *et al.*, 2003; Bellati *et al.*, 2014; Vasconcelos and de Sousa Mendes, 2015; Lawson and Rentea, 2022), but some studies have investigated fertility or parity after ART in women with previous UO (Lass *et al.*, 1997; Khan *et al.*, 2014; Lind *et al.*, 2018). A clinical multicentre study was previously conducted by our research group (Lind *et al.*, 2018) to specifically investigate that question. It included 22 847 infertile women treated with IVF/ICSI at five Swedish centres and found significantly fewer live births in the group of women with previous UO, when compared with controls lacking ovarian surgery. Our present findings investigating a large population-based cohort and using epidemiological research methods also indicate a negative impact of UO on future fertility, in line with our previous clinical study. The association between UO and negative IVF/ICSI outcomes is also supported by a recent meta-analysis (Rodriguez-Wallberg *et al.*, 2022).

The effect of UO on female reproduction has been elusive in the scientific literature. It is a well-known fact that the natural decline of the ovarian reserve due to oocyte atresia ends in infertility (Te Velde and Pearson, 2002), and thus, a surgery that removes half the ovarian reserve could be expected to have clinical implications on fertility. According to the conventional description of ovarian ageing, throughout life, there is a continuous reduction of the finite number of follicles pooled from both ovaries. In that model, the performance of a UO, which reduces the follicle pool by half, would have an age-dependent outcome where the ovarian age is drastically increased from the time of surgery. In an alternative model, presuming two separate follicular reserves, halving the ovarian pool would lead to follicle loss at half the speed compared to before UO. In the alternative model, UO would have only a small impact on reproductive lifespan, and the age at UO would be of little importance (Wilkosz *et al.*, 2014). A recent mathematical model proposed that the oversupply of primordial follicles at birth enables a simple stochastic mechanism for follicle activation, yielding a robust steady supply of growing and ovulating follicles for several decades (Lawley and Johnson, 2023). But none of these models can be fully matched to what is observed *in vivo* after UO, and we lack a prediction model for ovarian ageing that includes the observed compensatory mechanisms occurring when an ovary is suddenly lost (Wilkosz *et al.*, 2014).

To our knowledge, four studies have indicated a measurable effect of UO on the female reproductive life span, with an earlier menopausal age of approximately 1–2 years (Hardy and Kuh, 1999; Yasui *et al.*, 2012; Bjelland *et al.*, 2014; Rosendahl *et al.*, 2017). Only one of the studies reported on the effect of age at UO and indicated that the age at menopause was increasingly brought forward when UO was conducted at a younger age (Rosendahl *et al.*, 2017). Our data shows another aspect of the age-dependent effect of UO. Not only did exclusion of the age group of women 36–46 years have an impact on future childbirth among the nulliparous women with a change in RR from 0.63 (95% CI 0.61–0.65) to 0.80 (95% CI 0.77–0.82), but the likelihood of subsequent childbirth was also significantly reduced with increasing age at UO (≤25 years at time of UO (RR 0.89), 26–35 years (RR 0.81), ≥36 years at index date (RR 0.60)). The greater impact of UO among the older women could be a direct consequence of a shortening of time to menopause, as their natural decline in fertility has started, and surgery combined with earlier POI will drastically impact their remaining fertile window, more so than with a similar shortening of the reproductive lifespan among younger women. But, as the results remain significant for all age categories, we cannot exclude that UO also causes a reduction in fertility potential similar to what has been observed in women undergoing IVF, where the reduction of ovarian reserve after UO is believed to directly impact negatively the pregnancy rate (Lind *et al.*, 2018).

In the literature, there is also a documented correlation between bilateral oophorectomy and increased all-cause mortality (Shuster *et al.*, 2010; Rocca and Ulrich, 2012; Parker *et al.*, 2013), however, data on UO are scarce. In this study, we did not actively investigate cause of death after UO, but it is worth noting that the significant increase in mortality observed in women exposed to UO in our study, has also been documented in a previous American cohort study, in which similarly, an unexplained increase in mortality was observed among women under the age of 45 who had undergone UO for benign indications (Rivera *et al.*, 2009).

Our study is observational, and it also indicates that the chance of having future children also decreases with older age at the time of surgery, especially so among the women who were nulliparous at the time of UO. While the mechanisms behind this observed decrease in childbirth rate are unknown, our results suggest that unilateral oophorectomies should not be regarded as fertility-innocuous, especially among older women.

Further research is needed to clarify the underlying cause of the observed decrease in childbirth rate associated with undergoing UO for benign indications. We propose further studies to investigate the attempted pregnancy rate, the impact of underlying infertility, and the observed increase in all-cause mortality observed among the exposed cases.

A major strength of our study is the nation-wide population-based design, which has a high coverage, validity for surgical data (Ludvigsson *et al.*, 2011; National Board of Health and Welfare, 2011, 2003), and population diversity. Also, the use of the Swedish Patient Registry has allowed us to follow the entire cohort of women until the end of their fertile lifespan. A total number of 10 469 women with a unilateral oophorectomy were included, and matched with 101 753 controls, which renders our cohort study, with high-quality data, the largest up to date, with a sufficient power to detect significant differences. However, several limitations of our study should be acknowledged. First, we do not know if the desire for pregnancy was the same in both cases and controls; thus, we have made the assumption that the size of the cohort will balance the number of women attempting pregnancy in the respective groups, and a similar assumption was made for unknown infertility causes, including male factor infertility. Although the reduced likelihood of childbirth was greater in nulliparous women at the time of UO than in the full cohort (RR 0.63; 95% CI 0.61–0.65 vs RR 0.89; 95% CI 0.86–0.91), the fact that the cohorts were not matched for parity reduced the number of matched controls in the subgroup analyses. Also, the data did not allow us to adjust for all secondary factors possibly affecting fertility. To address the effect of underlying infertility, we did a sensitivity analysis excluding all women with a registered diagnosis of endometriosis (this was 18% of the cases and 0.003% of the controls), as well as a sensitivity analysis excluding any women undergoing infertility treatments prior to the index. We also performed a sensitivity analysis on all outcomes with exclusion of women undergoing further gynaecologic surgery, such as removal of the contralateral ovary, hysterectomy, and death. None of the sensitivity analyses significantly affected the association of UO to our primary outcome of reduced childbirth, nor our secondary outcomes of infertility and infertility treatment. On the contrary, exclusion of women with previous infertility strengthened the association between UO and later female infertility from RR 1.73 (1.59–1.87) to RR 5.10 (3.95–6.59). However, a sensitivity analysis on outcomes does not eliminate the confounding effect of the underlying diagnosis. And, while the vast majority of UOs are usually indicated by diagnoses such as endometriosis, benign ovarian cysts, and benign tumours (Borgfeldt and Andolf, 1999; Laughlin-Tommaso *et al.*, 2014; Lawson and Rentea, 2022), it was not possible to access the histopathology diagnoses of the removed ovaries in this study, which means that other possible diagnoses associated with a lower subsequent likelihood of childbirth could not be used as potential confounders. Additional limitations of the study included the lack of adjustment for lifestyle factors, as such data were only available from the medical birth register, and thus only accessible for the women who had given birth.

## Conclusions

Our findings are the first to show a significant association between UO for benign indications and a post-surgery decrease in the likelihood of future childbirth when following all women until the end of their reproductive life span. Our findings also suggest that the risk of not having future children is increased with older age at the time of surgery, especially among the women who were nulliparous at the time of UO. While the limitations of the study do not allow us to explore the underlying cause for the observed reduction in childbirth, and causality cannot be inferred from these observational data, our results support other available data on UO and fertility outcomes, indicating that unilateral oophorectomies should not be regarded as fertility innocuous. The association between UO and reduced childbirth suggests that women who are planned to undergo UO would benefit from fertility counselling prior to the procedure, especially when naturally approaching the end of their reproductive life span.

## Supplementary Material

hoag041_Supplementary_Data

## Data Availability

The data underlying this article were accessed from the Swedish population-based registers. The derived data generated in this research will be shared on reasonable request to the corresponding author.
