# Supplementary material for: Likelihood of childbirth in women with one versus two ovaries: a Swedish population-based study of women treated with unilateral oophorectomy for benign indications
Source: Hum Reprod Open. 2026 May 25;2026(3):hoag041. doi: 10.1093/hropen/hoag041 (PMC13234452; doi:10.1093/hropen/hoag041)
Supplement: hoag041_Supplementary_Data [file hoag041_supplementary_data.docx]

| **Variable** | **Usage** | **ICD-10, ICD-9, ICD-8 Codes, ICD-07**  **surgery procedure codes** | **Source** | **Time Period** |
| --- | --- | --- | --- | --- |
| Surgery or medical treatment for extrauterine pregnancy | Secondary outcome | 7190, 7100, 7101, 7112,7122, 7096, 7191, 7840  LBC10, LBC21, LBC97, LBD01, LBC11, LBE01,  LBC07 | National Patient Register | >2 months after index (surgery for unilateral oophorectomy UO) |
| Surgery due to infertility | Secondary outcome | 7192,7140,7141,  7142,7143,7144,  7145  LBF01, LBF00, LBF03, LBF20-70, LBF96, LBF31, LBF41, LBF51, LBF61 | National Patient Register | >12 months after index (surgery for UO) |
| IVF treatment | Secondary outcome | 7198,7285,7083,  7082  LBF11, LCA30, LAA10,LAA11, LBF97 | National Patient Register | >12 months after index (surgery for UO) |
| Complications registered by IVF | Secondary outcome | N98 (all) | National Patient Register | >12 months after index (surgery for UO) |
| Female infertility | Secondary outcome | 628, 634  646D  N969, N97 (all), | National Patient Register | >12 months after index (surgery for UO) |

**Supplementary Table S1 Secondary outcome variables retrieved and the corresponding ICD codes**

UO, unilateral oophorectomy

**Supplementary Table S2 Previous infertility and endometriosis, surgery, death and number of children post index.**

|  | | All women | | | Nulliparous women prior to index | | |
| --- | --- | --- | --- | --- | --- | --- | --- |
| **n missing** | **Variable** | **Case** | **Control** | **p-value^2^** | **Case** | **Control** | **p-value^2^** |
| 0 | Hysterectomy |  |  | <0.001*** |  |  | <0.001*** |
|  | No | 9,320 (89.0%) | 97,313 (95.6%) |  | 3,642 (89.2%) | 17,931 (95.5%) |  |
|  | Yes | 1,149 (11.0%) | 4,440 (4.4%) |  | 441 (10.8%) | 839 (4.5%) |  |
| 0 | Bilateral oophorectomy |  |  | <0.001*** |  |  | 0.001*** |
|  | No | 10 396 (99.3%) | 100,424 (98.7%) |  | 4,054 (99.3%) | 18,521 (98.7%) |  |
|  | Yes | 73 (0.7%) | 1,329 (1.3%) |  | 29 (0.7%) | 249 (1.3%) |  |
| 0 | Unilateral oophorectomy |  |  | <0.001*** |  |  | <0.001*** |
|  | No | 9,550 (91.2%) | 101,658 (99.9%) |  | 3,281 (90.9%) | 18,760 (99.9%) |  |
|  | Yes | 919 (8.8%) | 95 (0.1%) |  | 373 (9.1%) | 10 (0.1%) |  |
| 0 | Death |  |  | <0.001*** |  |  | <0.001*** |
|  | No | 10,041 (95.9%) | 99,892 (98.2%) |  | 3,874 (94.9%) | 18,363 (97.8%) |  |
|  | Yes | 428 (4.1%) | 1,861 (1.8%) |  | 209 (5.1%) | 407 (2.2%) |  |
| 0 | Endometriosis |  |  | <0.001*** |  |  | <0.001*** |
|  | No | 8,542 (81.6%) | 101,423 (99.7%) |  | 3,138 (76.9%) | 18,737 (99.8%) |  |
|  | Yes | 1,927 (18.4%) | 330 (0.3%) |  | 945 (23.1%) | 33 (0.2%) |  |
| 0 | Previous infertility |  |  | <0.001*** |  |  | <0.001*** |
|  | No | 9,846 (94.0%) | 98,241 (96.5%) |  | 3,623 (88.7%) | 14,682 (78.2%) |  |
|  | Yes | 623 (6.0%) | 3,512 (3.5%) |  | 460 (11.3%) | 4,088 (21.8%) |  |
| 0 | Number of children |  |  | <0.001*** |  |  | <0.001*** |
|  | =>3 | 434 (4.1%) | 4,490 (4.4%) |  | 363 (8.9%) | 2,982 (15.9%) |  |
|  | 0 | 7,804 (74.5%) | 72,585 (71.3%) |  | 2,396 (58.7%) | 6,381 (34.0%) |  |
|  | 1-2 | 2,231 (21.3%) | 24,678 (24.3%) |  | 1,324 (32.4%) | 9,407 (50.1%) |  |

Deaths, number of children and surgical events post index registered until the age of 46 years of age.

^2^ Significance as indicated by a chi-square test, ns: p>0,05 (Not significant); *: p ≤0.05 (Significant); ** p ≤0.01 (Highly significant); ***: p ≤0.001 (Highly significant)

**Supplementary Table S3 Sensitivity analysis on the association between unilateral oophorectomy and parity after exclusion of women who had a hysterectomy or bi- or unilateral oophorectomy, or who died, post index.**

|  | **All women N= 100,652** | | | | **Nulliparous women prior to index N= 20,470** | | | |
| --- | --- | --- | --- | --- | --- | --- | --- | --- |
| **Variable** | **Cases with UO**  **N=9,971**  **(%)** | **Controls**  **N= 90 681**  **(%)** | **RR (95% CI)** | **p-value^2^** | **Cases with UO**  **N= 3,856**  **(%)** | **Controls**  **N= 16,614**  **(%)** | **RR (95% CI)** | **p-value^2^** |
| One or more children born | 2,578 (25.8) | 26,067 (28.8) | 0.90 (0.88 to 0.92) | <0.0001*** | 1,637 (42.5) | 11,131 (67.0) | 0.63 (0.61 to 0.66) | <0.0001*** |
| Female infertility | 563 (5.6) | 2,908 (3.2) | 1.76 (1.62 to 1.92) | <0.0001*** | 418 (10.8) | 1,390 (8.4) | 1.30 (1.17 to 1.43) | <0.0001*** |
| Surgery for infertility | 123 (1.2) | 422 (0.5) | 2.65 (2.18 to 3.23) | <0.0001*** | 97 (2.5) | 233 (1.4) | 1.79 (1.42 to 2.27) | <0.0001*** |
| IVF treatment^a^ | 44 (0.4) | 150 (0.2) | 2.67 (1.91 to 3.73) | <0.0001*** | 40 (1.0) | 74 (0.4) | 2.33 (1.59 to 3.42) | <0.0001*** |

The association between unilateral oophorectomy (UO) and parity (children born) was investigated after exclusion of women who had a hysterectomy, bi or unilateral oophorectomy, or died after index date. Infertility diagnosis, surgery for infertility, and performance of infertility treatments with IVF post index are also indicated in each group.

^a^ IVF treatments: only women with index date from 1980 and onwards

^2^ Significance as indicated by a Generalized Estimating Equation (GEE) model with binomial distribution, ns: p>0.05 (Not significant); *: p ≤0.05 (Significant); ** p ≤0.01 (Highly significant); ***: p ≤0.001 (Highly significant)

**Supplementary Table S4. Sensitivity analysis on the association between unilateral oophorectomy and parity after exclusion of women older than 35 years at index.**

|  | **All women N= 55,921** | | | | **Nulliparous women prior to index N= 19,073** | | | |
| --- | --- | --- | --- | --- | --- | --- | --- | --- |
| **Variable** | **Cases with UO**  **N=5,180**  **(%)** | **Controls**  **N= 50,741**  **(%)** | **RR (95% CI)** | **p-value^2^** | **Cases with UO**  **N= 2,734**  **(%)** | **Controls**  **N= 16,339**  **(%)** | **RR (95% CI)** | **p-value^2^** |
| One or more children born | 2,501 (48.3) | 27,306 (53.8) | 0.90 (0.87 to 0.92) | <0.0001*** | 1,629 (59.6) | 12,216 (74.8) | 0.80 (0.77 to 0.82) | <0.0001*** |
| Female infertility | 540 (10.4) | 3,087 (6.1) | 1.71 (1.57 to 1.87) | <0.0001*** | 410 (15.0) | 1,553 (9.5) | 1.58 (1.43 to 1.74) | <0.0001*** |
| Surgery for infertility | 128 (2.5) | 488 (1.0) | 2.57 (2.12 to 3.11) | <0.0001*** | 102 (3.7) | 272 (1.7) | 2.24 (1.79 to 2.80) | <0.0001*** |
| IVF treatment^a^ | 39 (0.8) | 132 (0.3) | 2.89 (2.03 to 4.13) | <0.0001*** | 35 (1.3) | 76 (0.5) | 2.75 (1.85 to 4.10) | <0.0001*** |

The association between unilateral oophorectomy (UO) and parity (children born) was investigated after exclusion of women older than 35 years at index date. Infertility diagnosis, surgery for infertility, and performance of infertility treatments with IVF post index are also indicated in each group.

^a^ IVF treatments: only women with index date from 1980 and onwards are included.

^2^ Significance as indicated by a Generalized Estimating Equation (GEE) model with binomial distribution, ns: p>0.05 (Not significant); *: p ≤0.05 (Significant); ** p ≤0.01 (Highly significant); ***: p ≤0.001 (Highly significant)

**Supplementary Table S5. Sensitivity analysis on the association between unilateral oophorectomy and parity after exclusion of women with infertility treatment before index.**

|  | **All women N= 108,087** | | | | **Nulliparous women prior to index N= 18m305** | | | |
| --- | --- | --- | --- | --- | --- | --- | --- | --- |
| **Variable** | **Cases with UO**  **N=9,846**  **(%)** | **Controls**  **N= 98,241**  **(%)** | **RR (95% CI)** | **p-value^2^** | **Cases with UO**  **N= 3,623**  **(%)** | **Controls**  **N= 14,682**  **(%)** | **RR (95% CI)** | **p-value^2^** |
| One or more children born | 2,295 (23.3) | 26,647 (27.1) | 0.86 (0.83 to 0.88) | <0.0001*** | 1,415 (39.1) | 9,283 (63.2) | 0.62 (0.60 to 0.64) | <0.0001*** |
| Female infertility | 90 (0.9) | 176 (0.2) | 5.10 (3.95 to 6.59) | <0.0001*** | 61 (1.7) | 32 (0.2) | 7.72 (5.04 to 11.84) | <0.0001*** |
| Surgery for infertility | 39 (0.4) | 121 (0.1) | 3.22 (2.24 to 4.61) | <0.0001*** | 29 (0.8) | 43 (0.3) | 2.73 (1.71 to 4.37) | <0.0001*** |
| IVF treatment^a^ | 14 (0.14) | 35 (0.04) | 3.99 (2.15 to 7.41) | <0.0001*** | 12 (0.33) | 7 (0.05) | 6.94 (2.73 to 17.65) | <0.0001*** |

The association between unilateral oophorectomy (UO) and parity (children born) was investigated after exclusion of women with infertility treatment before index date. Infertility diagnosis, surgery for infertility, and performance of infertility treatments with IVF post index are also indicated in each group.

^a^ IVF treatments only women with index date from 1980 and onwards are included.

^2^ Significance as indicated by a Generalized Estimating Equation (GEE) model with binomial distribution, ns: p>0.05 (Not significant); *: p ≤0.05 (Significant); ** p ≤0.01 (Highly significant); ***: p ≤0.001 (Highly significant)
